# Repeatable Acoustic Vaporization of Coated Perfluorocarbon Bubbles for Micro-Actuation Inspired by *Polypodium aureum*

**DOI:** 10.3390/biomimetics9020106

**Published:** 2024-02-11

**Authors:** Se-Yun Jeong, Han-Bok Seo, Myung-Hyun Seo, Jin-Woo Cho, Seho Kwon, Gihun Son, Seung-Yop Lee

**Affiliations:** Department of Mechanical Engineering, Sogang University, Baekbeom-ro 35, Mapo-gu, Seoul 04107, Republic of Korea

**Keywords:** acoustic droplet vaporization, *Polypodium aureum*, perfluorocarbon, DDFP, contrast agent, micro actuator, microbot

## Abstract

*Polypodium aureum*, a fern, possesses a specialized spore-releasing mechanism like a catapult induced by the quick expansion of vaporized bubbles. This study introduces lipid-coated perfluorocarbon droplets to enable repeatable vaporization–condensation cycles, inspired by the repeatable vaporization of *Polypodium aureum*. Lipid-perfluorocarbon droplets have been considered not to exhibit repeatable oscillations due to bubble collapse of the low surface tension of lipid layers. However, a single lipid-dodecafluoropentane droplet with a diameter of 9.17 µm shows expansion–contraction oscillations over 4000 cycles by changing lipid composition and applying a low-power 1.7 MHz ultrasound to induce the partial vaporization of the droplets. The optimal combinations of shell composition, droplet fabrication, and acoustic conditions can minimize the damage on shell structure and promote a quick recovery of damaged shell layers. The highly expanding oscillatory microbubbles provide a new direction for fuel-free micro- or nanobots, as well as biomedical applications of contrast agents and drug delivery.

## 1. Introduction

In the past few decades, the precise fabrication and actuation of micro- or nano-sized objects has been actively developed for numerous fields such as biomedical imaging, therapy, and surgery [1,2,3], as well as nano- or micro actuators and robots [4,5,6]. Various power sources such as chemical fuels, acoustic waves, magnetic fields, light, or thermal energy have been considered for the actuation of small-scale robots. However, new alternative fuels and propulsion mechanisms are necessary for safe and sustainable operation in the human body [4].

**Figure 1:**
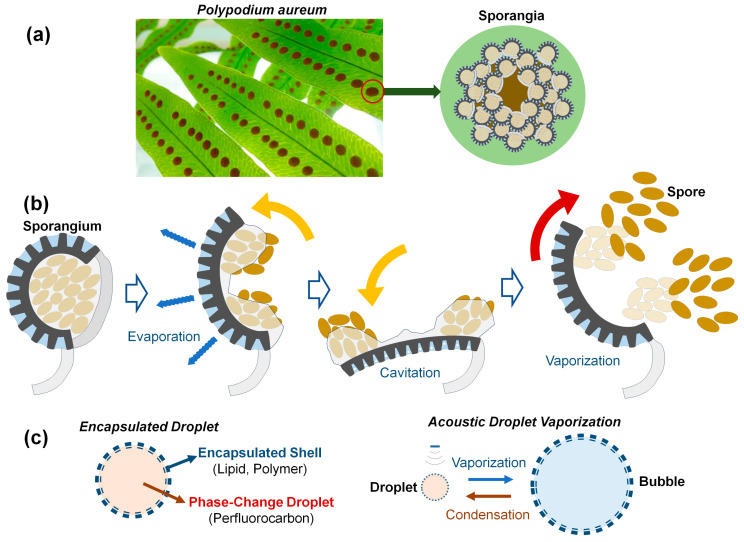
(**a**) Photo of a fern *Polypodium aureum* leaf containing the round, bright-orange clusters of sporangia. (**b**) Brief actuation mechanism of the sporangium to release spores based on slow evaporation and quick cavitation vaporization. Sporangium opens as the annulus cells lose water by evaporation. Cavitation within the annular cells causes vaporization, leading to fast closure and ejection of spores in 10~30 ms [9,10]. (**c**) Encapsulated droplets of shell-perfluorocabon (PFC) exhibit size variation due to acoustic droplet vaporization (ADV) and bubble condensation at low temperatures.

Numerous organisms in nature have efficiently functioning actuators across various sizes, serving as a great source of inspiration for technological advancements in human society. Various shooting mechanisms of spores and seeds in fungi and plants use water condensation, absorption, and vaporization [7]. A representative example is a fern, *Polypodium aureum*, belonging to the phelebodium genus (Figure 1a). *Polypodium aureum* possesses specialized sporangia for dispersing spores. The sporangium is composed of 12 to 13 annulus cells. As the annulus cells lose water by evaporation, water tension grows within them, thus causing the radial wall to shrink [8,9]. The force required to bend the annulus walls during opening is balanced by the negative pressure or water tension inside the cells. When the water tension is too large and the pressure falls below a critical threshold, cavitation bubbles are formed within several cells, leading to the fast closure and ejection of the spores like a catapult [10], as illustrated in Figure 1b. In this process, spores with a size of 300 µm are ejected at speeds of 10 m/s in just 10 µs [9]. The process of slow evaporation–quick vaporization could be repeated many times by placing the empty sporangium in water for a few minutes [10]. It is worth noting that the specific structure and material of the annulus cells for spore release in *Polypodium aureum* provide the repetitive expansion–contraction motion without internal fuel or power. This study suggests a possible direction for structural designs for self-powered micro actuators or a long-lasting contrast agent, inspired by the repeatable vaporization of *Polypodium aureum.*

Liquid droplets or gaseous bubbles undergo phase changes depending on the temperature, pressure, and medium conditions within a given system. As shown in Figure 1c, external stimuli such as ultrasound, laser, and heat sources can cause size variation in droplets and bubbles due to vaporization or condensation [1,2,3]. The acoustic droplet vaporization (ADV) technique, using phase-changing perfluorocarbon (PFC) droplets such as perfluoropentane (PFP), perfluorohexane (PFH), perfluorobutane (PFB), and perfluorooctane (PFO), has shown great potential for diverse biomedical applications including local drug delivery to target tissues [11]. There are two kinds of shell materials encapsulating phase change droplets. Hard shells use a coating of lower viscoelastic properties such as polymers or denatured proteins, as well as porous silica materials encapsulating gas. Generally, hard shell bubbles show an increased circulation time in vivo and are the preferred type of contrast agents for higher-intensity ultrasound applications [2]. Soft shells using lipids or surfactant molecules are commonly used for examinations using a low mechanical index since these soft-shell bubbles are sensitively detectable due to the thinner, more flexible materials compared with hard-shell ones, as well as the combination by hydrophobic interactions. Therefore, after slight shell disruptions, the shell reseals itself to minimize surface tension [12]. If sealing is not possible due to the high acoustic pressure, the microbubbles will split into several smaller bubbles, unlike the case of hard-shell microbubbles [2].

PFC droplets vaporize at a negative pressure well above the ADV threshold. One or ten cycles of acoustically induced vaporization–condensation of lipid-PFC droplets have been reported in [13,14]. Marmottant et al. [15] observed expansion–contraction oscillations of a lipid-PFB bubble over 60 cycles when the acoustic pulse frequency increased from 1.5 to 4 MHz using low acoustic power to avoid shell buckling and bubble rupture.

The lipid-PFC droplets have been considered to not exhibit repeatable expansion-contraction oscillations due to the low mechanical properties of lipid layers. Encapsulated PFC droplets that demonstrate a large number of vaporization–condensation cycles by ADV have not yet been reported in the literature. This study proposes a new fabrication method of lipid-PFC droplets to enable repeatable vaporization–condensation cycles for hundreds to thousands of times. The repeatable and stable oscillations are mainly achieved by changing the shell-layer composition and applying lower ultrasound powers for the partial vaporization of droplets to minimize damage on the shell structure and for the quick recovery of the damaged lipid layers.

## 2. Materials and Methods

### 2.1. Materials

Dodecafluoropentane (DDFP) from Synquest Labs (Alachua, FL, USA) was used as the core of PC droplets. DDFP has a boiling point of 29 °C at atmospheric pressure. The lipid shell encapsulating the PC droplet was fabricated using 1,2-ditearoyl-sn-glycero-3-phosphocholine (DSPC) and 1,2-dioctadecanoyl-sn-glycero-3-phosphoethanolamine-polyethylene glycol-2000 (DSPE-PEG2000), which were purchased from Avanti Polar Lipids (Alabaster, AL, USA).

DSPC and DSPE-PEG2000 were dissolved in chloroform at molar ratios of 9:1 or 7:3 in a 2 mL clear vial. The vial was placed in a vacuum chamber for 12 h to completely remove the chloroform. Subsequently, a thin white lipid film formed at the bottom of the vial was hydrated with 1.8 mL of distilled water and sonicated using a bath sonicator (Powersonic 505, Hwashin Instrument, Seoul, Republic of Korea) at 56 °C. After allowing the vial to rest with the lipid solution at room temperature, liquid DDFP was added to the vial to achieve a volume fraction of 10% [16]. Finally, DDFP droplets were created by agitating the vials for 45 s in a Vialmix (Lantheus Holdings, Billerica, MA, USA).

### 2.2. Experimental Setup

**Figure 2:**
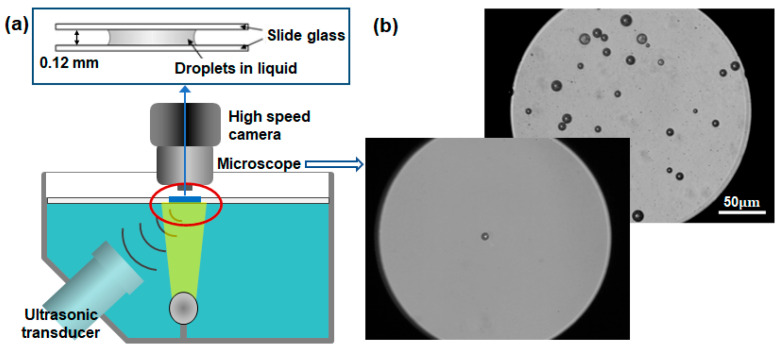
(**a**) Experimental setup composed of an ultrasonic transducer of 1.074 MHz and a high-speed camera connected to an inverted microscope to capture lipid-DDFP droplets. (**b**) Microscopic images of micro-sized lipid-DDFP droplets.

Figure 2 illustrates the current experimental arrangement. A clear acrylic water tank measuring 0.3 m × 0.2 m × 0.1 m was filled with degassed water and positioned atop an inverted microscope (Epiphot 200, Nikon, Minato, Japan). Within the water tank, a submersible heater was installed to maintain the water temperature at either 24 °C or 36 °C. To excite lipid-DDFP droplets, a high-intensity focused ultrasound (HIFU) transducer (HFT55M576, EofE Ultrasonics, Goyang, Republic of Korea) with a focal length of 35 mm and a diameter of 40 mm was employed at a central frequency of 1.05 MHz. The acoustic pressure amplitude and excitation time were precisely regulated using a function generator (33500B, Keysight, Santa Rosa, CA, USA). A manual trigger was used to provide a single pulse of 1.05 MHz HIFU for one cycle of vaporization–recondensation. For the repeatable vaporization and condensation experiments, a disc-type ultrasound device was self-manufactured to provide an ultrasonic frequency of 1.7 MHz with adjustable powers, which was used to generate high-density nanobubbles [17].

An inverted microscope was equipped with a high-speed camera (VEO 410, Phantom, Wayne, NJ, USA), enabling sample rates ranging from 57,000 fps (17.54 µs) at a resolution of 256 × 256 pixels up to 250,000 fps (4 µs) at 128 × 64 pixels. This high-speed camera was synchronized with a function generator, ensuring that images were simultaneously captured via manual triggering. To magnify samples larger than 1 μm in diameter, a 10× objective lens (PLN10X/0.25, Olympus, Shinjuku, Japan) and a 50× lens (CF Plan 50 × 0.55 EPI ELWD, Nikon, Japan) were employed. The droplet samples were sandwiched between two glass slides for the precise observation of their vaporization and condensation. A microstage positioned the active transducer at the location of highest ultrasonic pressure. Once proper alignment had been achieved, both the inverted microscope and transducer were securely affixed to the optical table. The droplet samples intended for testing were meticulously focused optically and acoustically by adjusting a water tank, facilitated by a linear stage integrated into the microscope.

## 3. Results and Discussion

### 3.1. Recondensation after Vaporization and Bubble Collapse

**Figure 3:**
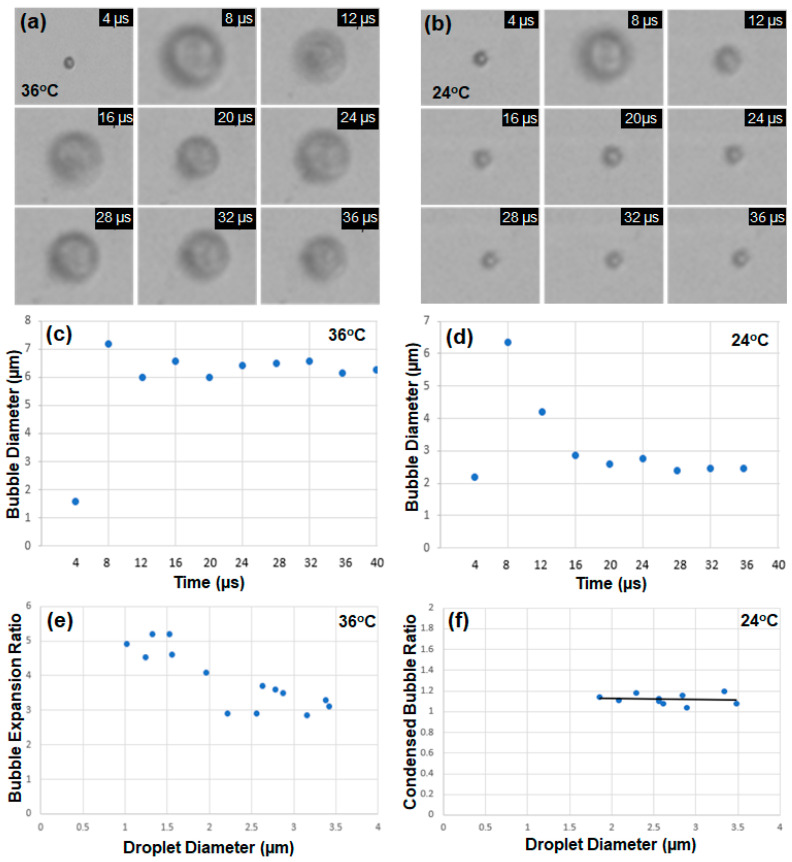
(**a**) The timeline images of a single droplet with an initial diameter of 1.6 μm, excited by an ultrasound pulse of 1 μs and power of 10 W with peak rarefactional pressure of 2.1 MPa at 36 °C. (**b**) The timeline images of a single droplet with an initial diameter of 2.2 μm at 24 °C. (**c**) The diameter of a droplet increased from 1.6 to 7.2 μm at 36 °C with an ultrasound pulse of 1 μs. (**d**) The droplet with an initial diameter of 2.2 μm exhibited vaporization and recondensation at 24 °C with an ultrasound pulse. (**e**) The bubble expansion ratios of fourteen droplet samples with diameters between 1.5 and 3.5 µm with acoustic vaporization at 36 °C. (**f**) The bubble diameter variations before vaporization and after recondensation of nine droplet samples at 24 °C.

Encapsulated droplets with diameters between 1.5 and 3.5 µm were tested to observe vaporization and recondensation. Figure 3a shows the timeline images of a single droplet with an initial diameter of 1.6 μm captured at every 4 μs when an ultrasound pulse of 1 μs at HIFU power of 10 W with the peak rarefactional pressure of 2.1 MPa was applied, and the water temperature was kept at 36 °C. The vaporized bubble kept expanding at 36 °C, which is above the boiling point (29 °C) of DDFP. However, when the temperature was set to be 24 °C below the boiling point (29 °C), the droplet with an initial diameter of 2.2 μm exhibited expansion by vaporization and then contraction by recondensation within 8 μs, as shown in Figure 3b.

Figure 3c,d indicate the timeline of the bubble diameter at 36 °C and 24 °C, respectively. The bubble diameter increased from 1.6 to 7.2 μm (approximately 4.5 times) at 36 °C. The overshoot of transient shrinking and the expanding motion observed after the first expansion at 8 μs indicates that the encapsulated shell composed of the lipid (DSPC) and the lipopolymer (DSPE-PEG2000) had elastic material properties. The diameter of the coated droplet in Figure 3d increased from 2.2 to 6.3 μm (approximately 2.9 times) due to vaporization and from 2.2 to 2.4 μm (approximately 1.1 times) after the vaporization–recondensation cycle. Fourteen droplet samples with diameters between 0.5 and 3.5 µm were tested at 36 °C in Figure 3e. The expansion ratio decreased slightly with increasing droplet size, and the maximum ratio was 5.2. Similarly, nine droplet samples with diameters between 1.5 and 3.5 µm were tested at 24 °C, and the bubble diameter variations before vaporization and after recondensation were measured. The average expansion ratio of the samples was 1.12 (Figure 3f).

**Figure 4:**
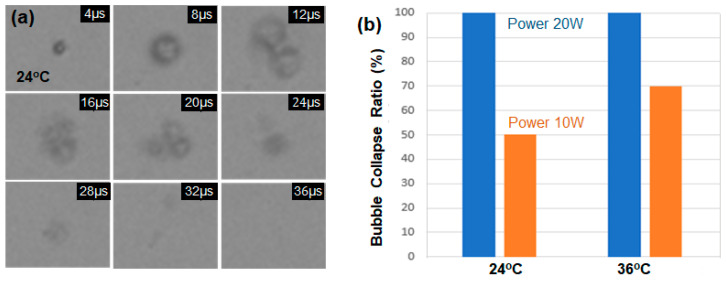
(**a**) The timeline images of a single droplet, excited by an ultrasound pulse of 1 μs and power of 20 W with the peak rarefactional pressure of 4.2 MPa at 24 °C. (**b**) The bubble collapse ratio depending on the acoustic power (10 W and 20 W) and the temperature (24 and 36 °C).

In the previous experiments, droplet samples exposed to an ultrasound power of 10 W with the peak rarefactional pressure of 2.1 MPa disappeared due to shell collapse during vaporization. To compare the shell collapse ratio by input power, the ultrasound power was increased to 20 W (4.2 MPa), and Figure 4a shows the collapsed motion of a single droplet sample at 24 °C. All encapsulated droplet samples disappeared at the power of 20 W. At high acoustic pressures, the droplet undergoes forced expansion and compression, which results in its destruction by either outward diffusion of the vaporized gas during the compression phase or diffusion via large shell defects, or by fragmentation [18]. Figure 4b compares the bubble collapse ratio at the power of 10 W and 20 W among the ten droplet samples at 24 and 36 °C, respectively. The bubble survival rates without shell collapse at the power of 10 W are 50% and 30% for the cases of 24 and 36 °C, respectively. When 20 W pulses of ultrasound of 1.07 MHz were excited into the droplet samples at 24 °C, all the samples disappeared without repetitive vaporization and condensation cycles. It is well known that the ADV threshold is inversely correlated with droplet size [11], and it was recently reported that the temperature threshold for the recondensation of vaporized droplets also decreases with increasing droplet size [19].

### 3.2. Repetitive Vaporization–Condensation

One or tens cycles of acoustically induced vaporization–condensation of lipid-PFC droplets have been reported in [13,14,15]. The article [13] studied ADV dynamics containing three different formulations of PFC emulsions such as PFP, PFH, and PFO, observing that bubbles in PFO emulsions underwent repeated vaporization–recondensation or fragmentation. Namen et al. [14] demonstrated three consecutive vaporization–condensation cycles of nano-sized PFH droplets with lipid shells using 50 pulses of 10 cycles of HIFU and ultrasound B-mode image frames. The average peak diameter for the PFH samples was 558.71 ± 31.58 nm at an average concentration of 1.8 × 10^8^ particles/mL. Marmottant et al. [15] observed expansion–contraction oscillations of a lipid-PFB bubble with a boiling point of −2 °C over 60 cycles in 35 μs when the acoustic pulse frequency increased from 1.5 to 4 MHz using low acoustic power to avoid shell buckling and bubble rupture. The oscillations of the bubble diameter, which were within 30% of the initial diameter, exhibited expansion–contraction cycles that decreased with increasing acoustic frequency and oscillation cycle.

Optically, vaporization–condensation cycles have been reported using laser pulses [19]. In the report, indocyanine green (ICG)-loaded PFP droplets with bovine serum albumin (BSA) shells were locally heated using laser input. Six vaporization–condensation cycles of 2.0 μm droplets were observed in response to six consecutive short-pulse laser excitations (5 ns duration, 800 nm wavelength) using a high-speed camera of 5,000,000 fps. It is noted that the droplet size remained unchanged after one hour post exposure of six consecutive laser excitations, indicating the stability of the 2.0 μm droplet. However, some larger droplets (>7.5 μm) tended to grow continually into a large gas bubble after undergoing a single vaporization and contraction event. The bubble instability prevented repetitive optical droplet vaporization (ODV). Moreover, the optical droplet vaporization of encapsulated PFC droplets via pulsed laser absorption limited the imaging depth and temporal resolution for biomedical applications.

The repeatable vaporization–condensation of PFC droplets by acoustic excitations offers several benefits such as longer circulation times, extended imaging windows and higher sensitivity, as well as higher imaging depth and resolution [14,20]. Encapsulated PFC droplets to demonstrate a large number of vaporization–condensation cycles by ADV have not yet been reported in the literature. Several design parameters are involved in the mechanical properties and stability of the shell layers and the threshold of PFC droplets. Ultrasound frequency, power, pulse duration, and focal distance to droplet samples are carefully controlled to minimize the collapse of vaporized bubbles.

The repeatable vaporization–condensation tests were implemented under similar conditions to the previous one-cycle vaporization–condensation experiments in Figure 4b, where the ratio of the lipid (DSPC) and the lipopolymer (DSPE-PEG2000) was 9:1, and the ultrasound power was 10 W at 1.05 MHz under a temperature of 24 °C. However, all the coated DDFP micro-droplets collapsed and disappeared after one or a few ultrasonic cycles. Several researchers have studied the effect of varying the concentration of DSPE-PEG2000 with respect to DPPC (1,2-dipalmitoyl-sn-glycero-3-phosphocholine) on the intensity and duration of PFC droplets [21,22]. Welch et al. [21] reported the size variation and duration of nanodroplets of PFP and PFH combinations for the cases of different DPPC and DSPE-PEG2000 ratios (9:1, 5:5, and 1:9). PFC nanodroplets with a lipid shell composed of a 5:5 ratio yielded the highest B-mode image intensity and duration. However, nanodroplets with a ratio of 1:9 had lower acoustic signal and duration because the addition of too much lipopolymer could create steric hindrance issues and create lipid shells too stiff for efficient nanodroplet expansion. The proper lipopolymer concentration in the shell composition provides the greatest pressure difference between the acoustic droplet vaporization (liquid to gas phase transition) threshold and inertial cavitation (rapid collapse of the vaporized droplets) onset [21]. Chattaraj et al. [22] also reported that increasing DSPE-PEG2000 concentration in nanodroplets from 3% mol to 20% mol caused a significant increase in acoustic image intensity.

Therefore, for the repeatable expansion experiments, the lipid composition ratio was decreased from 9:1 to 7:3 to alter the properties of the shell layers. Secondly, a disc-type ultrasonic transducer of 1.7 MHz providing low powers was self-manufactured, which was used to generate high-density nanobubbles [17].

**Figure 5:**
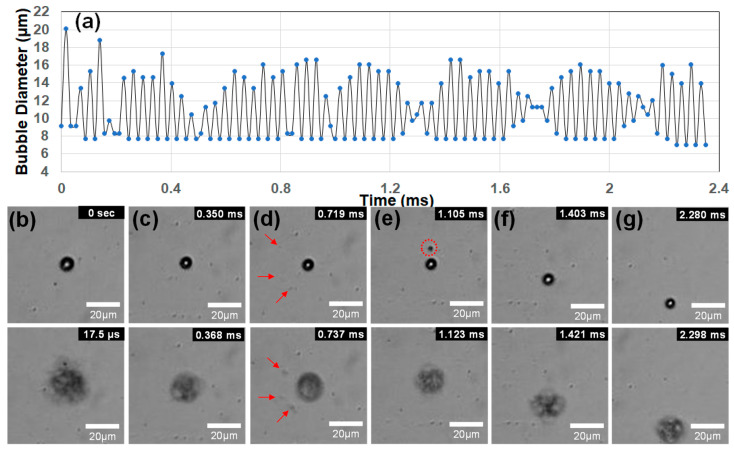
(**a**) A single lipid-DDFP droplet with an initial diameter of 9.17 µm exhibited 62 consecutive vaporization–condensation cycles using 1.7 MHz ultrasound and a sampling time of 17.54 µs at 24 °C. (**b**–**g**) Six vaporization–condensation oscillation images of the droplet sample. After the first vaporization, the bubble size decreased slightly with increasing cycles. Bubble fragmentation was observed to occur due to the expanding and shrinking oscillations in (**d**,**e**). The three droplet fragments in (**d**), indicated by the red arrows, expand individually without merging back into the main droplet. However, a single fragment in (**e**) does merge during the subsequent vaporization.

For the shell composition with the 7:3 ratio of DSPC and DSPE-PEG2000, the disc-type ultrasound transducer of 1.7 MHz frequency generated stable vaporization and recondensation motions at the low power of 3 W with a peak rarefactional pressure of 0.96 MPa. Figure 5a shows 62 consecutive vaporization–condensation cycles of a single lipid-coated DDFC droplet with an initial diameter of 9.17 µm using 1.7 MHz ultrasound. The bubble diameter of each image frame was measured using open-source software ImageJ (www.imagj.net). The water temperature was kept at 24 °C. To observe the continuous movement of the excited droplet bubble, the frame rate of the high-speed camera was set to be 57,000 fps (17.54 µs) at a resolution of 256 × 256 pixels. The droplet sample showing the highest cycles could not be measured after 62 oscillation cycles (2.35 ms) because the bubble moved out of the focal image boundary rather than collapsing. The ultrasound frequency of 1.7 MHz imposed consecutive positive–negative pressure oscillations on the droplet with a period of 0.588 μs. The experimental results [15], which report 60 oscillation cycles of a lipid-PFB bubble in 35 μs using an ultra-speed camera of 15 Mfps (66.7 ns), clearly show that the gas bubble exhibited oscillatory motions responding to every oscillation of frequencies from 1.5 MHz to 4 MHz ultrasound. Therefore, the lipid-DDFP droplet in Figure 5a indicated vaporization–condensation oscillations of 4000 cycles (=2.35 ms/0.558 μs) by responding to every oscillation of 1.7 MHz ultrasound, and it represented the highest number of vaporization oscillations of lipid-PFC droplets published in the literature to date.

The first vaporization image at 17.54 µs has the largest diameter, of 20.14 µm, over all the cycles, and the maximal expansion ratio is 2.2, which is lower than the ratio of 4.5 by vaporization in Figure 3c or the ratio of 2.9 in one cycle of vaporization–recondensation in Figure 3d. Therefore, it seems that the lower ultrasonic power leads to the partial vaporization of the droplet, resulting in minimized damage on the shell structure, stable vaporization–condensation motions, and reduced oscillation amplitude. The minimum diameters changed little at the condensation stages. The nonlinear oscillations with lower contraction amplitudes in Figure 5a ensured the recondensation of the vaporized bubble since the oscillatory amplitude of the condensed droplet was much smaller than that of the vaporized bubble.

Figure 5b–g show six different vaporization–condensation images of the single droplet. After the first vaporization, the bubble size decreases slightly with increasing cycles. When slight shell disruptions occur during expansion, the shell seals itself to minimize surface tension [12]. If sealing is not possible due to the high acoustic pressure, the expanding bubble will split into several smaller fragmentation bubbles instead of bursting like hard-shell encapsulated droplets [2]. As illustrated in Figure 5d,e, bubble fragmentation was observed to occur due to the expanding and shrinking oscillations. It is also known that fragmentations become complete encapsulated droplets consisting of a lipid shell and PFC core [18]. Lipid-coated three fragments in Figure 5d are located away from the main droplet, and they expand individually without coalescence. On the other hand, a single fragment in Figure 5e merges into the main bubble during the subsequent vaporization. Small split fragmentations were observed in 9.6% of the 135 image frames captured with a time interval of 17.54 µs. The highly repeatable oscillations of the micro-droplet ensured that most of the fragmentations underwent coalescence during the additional vaporizations of the main droplet, and the shedding of lipids and dissolution of the PFC core were minimized.

### 3.3. Future Direction for Micro-Sized Actuation and Biomedical Applications

**Figure 6:**
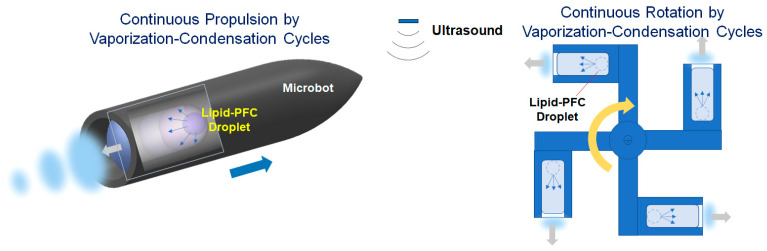
Concepts of linear and rotational micro-actuators using repeatable vaporization–condensation cycles of lipid-coated PFC bubbles.

Various types of microbots or nanobots based on distinct actuation principles using chemical fuels, acoustic waves, magnetic fields, light, or thermal energy have been developed in the past decade [4,5,6]. Ultrasound can realize noninvasive and on-demand motion control with long lifetime and good biocompatibility when it is used as an external energy input for micro actuators [6]. Moreover, acoustic actuation offers many advantages in terms of strong penetration, high flexibility, and good biocompatibility, making it highly desirable for many emerging fields [23]. Figure 6 shows two typical fields of micro actuators based on linear and rotational propulsions. Many types of acoustically driven actuators have been developed, such as acoustically propelled rod/wire/tube microbots, acoustically resonated microbubble actuators, acoustic motors/pumps, and acoustic tweezers [23,24,25,26]. Acoustic bubble actuators use the resonance behavior of trapped air bubbles, as the acoustic frequency approaches the resonance frequency of the bubbles [5]. However, the propulsion power is relatively small due to the low expansion ratio at resonance.

Micro actuators using phase-change droplets have been applied in short-time expansion and propulsion, such as through contrast agents [2,3], microbullets [27], and microcannons [28]. Thin lipid layers with low surface tension, as shell materials, are considered inadequate for use in repeatable expansion–contraction oscillations of phase-change bubbles. This study proposes a new direction for the biomedical applications of highly expanding oscillatory bubbles by demonstrating thousands of vaporization–condensation oscillations of lipid-DDFP droplets. However, there are still many problems to be solved for the use of long-time oscillating bubbles.

To improve the robust oscillations of lipid-coated PFC droplets, optimal combinations of design parameters such as shell composition and mixture ratio, droplet fabrication technique, acoustic frequency and power, and experimental setups are important. A variety of techniques have been developed for the fabrication of PFC droplets, such as agitation, sonication, extrusion, condensation, and microfluidic techniques [3]. In particular, shell materials and composition ratios that provide higher surface tension, lower gas leaking, and faster damage recovery should be developed to enhance the stability and durability of the coated droplets. These materials could include various lipids (DSPC, DMPC, DBPC) and lipopolymers (PEG40S, DSPE-PEG350/750/2000) [29,30] and new shell compounds of PEG-based polymers, including diblock copolymer constructs [4].

## 4. Conclusions

This study introduced lipid-coated DDFP droplets to enable repeatable vaporization–condensation cycles, inspired by the repeatable vaporization of *Polypodium aureum*. A single encapsulated DDFP droplet with a diameter of 9.17 µm exhibited expansion–contraction oscillations over 4000 cycles, which is the highest number of vaporization oscillations published in the literature to date. The highly repeatable microbubble was fabricated by using the lipid composition of the 7:3 ratio between DSPC and DSPE-PEG200, and it was excited by the low power (5 W) of the 1.7 MHz ultrasound to cause the partial vaporization of droplets and minimize damage to the shell structure. The highly expanding oscillatory microbubbles provide a new direction for fuel-free micro or nanobots as well as biomedical applications of contrast agents and drug delivery.

## Data Availability

The data that support the findings of this study are available from the corresponding author upon reasonable request.
